# TNF causes changes in glomerular endothelial permeability and morphology through a Rho and myosin light chain kinase‐dependent mechanism

**DOI:** 10.14814/phy2.12636

**Published:** 2015-12-03

**Authors:** Chang Xu, Xiaoyan Wu, Bradley K. Hack, Lihua Bao, Patrick N. Cunningham

**Affiliations:** ^1^ Section of NephrologyDepartment of MedicineUniversity of ChicagoChicagoIllinois; ^2^ Department of PediatricsBaylor College of MedicineHoustonTexas

**Keywords:** Acute kidney injury, cytokines, cytoskeleton, fenestrae, glycocalyx, inflammation

## Abstract

A key function of the endothelium is to serve as a regulated barrier between tissue compartments. We have previously shown that tumor necrosis factor (TNF) plays a crucial role in lipopolysaccharide (LPS)‐induced acute kidney injury, in part by causing injury to the renal endothelium through its receptor TNFR1. Here, we report that TNF increased permeability to albumin in primary culture mouse renal endothelial cells, as well as human glomerular endothelial cells. This process occurred in association with changes in the actin cytoskeleton and was associated with gaps between previously confluent cells in culture and decreases in the tight junction protein occludin. This process was dependent on myosin light chain activation, as seen by its prevention with Rho‐associated kinase and myosin light chain kinase (MLCK) inhibitors. Surprisingly, permeability was not blocked by inhibition of apoptosis with caspase inhibitors. Additionally, we found that the renal glycocalyx, which plays an important role in barrier function, was also degraded by TNF in a Rho and MLCK dependent fashion. TNF treatment caused a decrease in the size of endothelial fenestrae, dependent on Rho and MLCK, although the relevance of this to changes in permeability is uncertain. In summary, TNF‐induced barrier dysfunction in renal endothelial cells is crucially dependent upon the Rho/MLCK signaling pathway.

## Introduction

Acute kidney injury (AKI) is a frequent and serious complication of sepsis. The incidence of AKI is as high as 40% in patients with severe sepsis and septic shock. Moreover, there is strong evidence that AKI in patients with severe sepsis is associated with a higher mortality rate (Oppert et al. 2008). The high frequency and mortality of sepsis‐associated AKI demand a better understanding of the pathophysiology of this disorder (Muntner and Warnock 2010; Zarjou and Agarwal 2011).

Various studies have demonstrated, using the lipopolysaccharide (LPS) model of sepsis, that the cytokine tumor necrosis factor (TNF) plays a key, causative role in AKI through its action on renal endothelial TNFR1 (Al‐Lamki et al. 2001; Knotek et al. 2001; Cunningham et al. 2002; Xu et al. 2014). We have previously demonstrated that TNF‐induced inflammation in renal endothelial cells is enhanced by a Rho and myosin light chain kinase (MLCK)‐dependent mechanism (Wu et al. 2009). Rho‐associated kinase (ROCK) and MLCK are activated by a wide variety of extracellular stimuli, including cytokines such as TNF. ROCK phosphorylates and inhibits myosin light chain phosphatase; this event, along with MLCK activation, promotes myosin light chain (MLC) phosphorylation, which causes the assembly of actin filaments and actomyosin contractility (Petrache et al. 2003; Birukova et al. 2004; Wadgaonkar et al. 2005; Graham et al. 2006). These cytoskeletal changes, in turn, may greatly influence inflammatory signaling (Wadgaonkar et al. 2005; Wu et al. 2009).

The Rho and MLCK pathways are also known to be a key mediator of increased endothelial permeability in various endothelial cell types. Cytokines released during sepsis cause some of the most frequent clinical features of this syndrome, such as hypotension, edema, and hypoalbuminemia, in part through their actions on endothelium. A key function of the endothelium is to serve as a regulated barrier partially separating the contents of the blood from the extravascular space. Various stimuli such as thrombin, LPS, and also TNF have been shown to increase endothelial permeability to macromolecules in cell lines such as HUVECs and pulmonary capillary endothelial cells in part through Rho and MLCK pathways (Birukova et al. 2004; McKenzie and Ridley 2007). However, there is great diversity of the microvasculature in various organs (Aird 2003). In contrast to most other vascular beds, the highly specialized renal capillary endothelium is relatively permeable at baseline, containing fenestrae in the glomerular and peritubular capillaries which allow first the filtration, and then the significant resorption a high volume of fluid (Haraldsson et al. 2008; Satchell and Braet 2009).

Tumor necrosis factor has also been reported to cause disruption of the glycocalyx which lines the endothelium (Henry and Duling 2000). The glycocalyx layer consists of a wide variety of endothelial membrane‐associated macromolecules (Pries et al. 2000; Curry and Adamson 2012). These include the very negatively charged glycoproteins‐bearing acidic oligosaccharides with terminal sialic acids, and negatively charged proteoglycans with their associated glycosaminoglycan side chains such as heparan sulfate and chondroitin sulfate. These cell surface‐anchored molecules associate with adsorbed plasma components, including albumin, in dynamic equilibrium with the flowing blood in the lumen. This structure plays a key role in the regulation of vascular permeability, and is known to be degraded by ischemic and inflammatory stress (Pries et al. 2000; Reitsma et al. 2007; Becker et al. 2010). Recently, significant degradation of the glomerular glycocalyx in the setting of sepsis has been reported, in association with albuminuria (Lygizos et al. 2013; Xu et al. 2014).

The aim of this study was therefore to better define the role of Rho and MLCK in the endothelial injury and increased permeability induced by TNF in the specialized renal endothelium.

## Methods

### Antibodies and reagents

Wheat Germ Agglutinin (WGA) Alexa Fluor^®^ 594 Conjugate, Alexa Fluor^®^ 594‐Phalloidin, Alexa Fluor^®^ 488‐Phalloidin, and Abs to ZO‐1, occludin, and claudin‐5, were purchased from Life Technologies (Grand Island, UY); Claudin‐15 Ab was a gift from Dr Mikio Furuse (Kobe University, Japan); Claudin‐12 Ab from Santa Cruz Biotechnology (Santa Cruz, CA); Heparanase Ab from ProSpec (East Brunswick, NJ); and heparan sulfate proteoglycan Ab from US Biological (Marblehead, MA). Y‐27632, ML‐7, SB202219, and SP600125 were from EMD Millipore (Billerica, MA). TNF was from Peprotech (Rocky Hill, NJ). Broad‐ spectrum caspase inhibitors, z‐VAD‐fmk and boc‐D‐fmk, were from MP Biomedicals (Solon, OH).

### Evans blue dye leak assay

Male C57BL/6 mice of 10 weeks age were injected with 0.25 mg *Escherichia coli* LPS, serotype 0111:B4 (Sigma Chemical, St. Louis, MO) under isoflurane anesthesia. Twenty‐three hours after LPS, mice were injected with 1% EBD albumin conjugate via carotid venous catheter at a dose of 2 mL/kg. One hour later, laparotomy was performed and kidneys were perfused at the aorta with 10 mL PBS‐containing 0.05 mol/L citrate (pH 3.5) at a rate of 2.5 mL/min by a syringe pump. Kidneys were then removed, briefly dried using tissue paper, and weighed. Kidneys were immersed in formamide, homogenized, and incubated at 60°C overnight. Homogenates were centrifuged at 12,000 *g* for 30 min. The optical density of EBD in supernatant was determined at 620 and 740 nm. EBD concentration was calculated against a standard curve using the formula: OD620 (EBD) = OD620 − (1.1649 × OD740 + 0.004), and expressed as *μ*g EBD/g wet kidney weight. All animal experiments were performed under a protocol approved by the Institutional Animal Care and Use Committee.

### Cell culture and inhibitors

Primary culture of REnCs were prepared from mouse kidneys as described (Wu et al. 2009) and were grown in DMEM (Life Technologies) supplemented with 20% fetal bovine serum (FBS), 1% nonessential amino acids, 20 mmol/L HEPES, 2 mmol/L sodium pyruvate, 0.5 g/L d‐valine, heparin (100 *μ*g/mL), endothelial cell growth supplement (100 *μ*g/mL), 100 U/mL penicillin, and 100 mg/mL streptomycin. Human GEnCs were purchased from Applied Cell Biology Research Institute (Kirkland, WA) and grown in identical media.

### Ligase‐mediated‐PCR for apoptosis

Genomic DNA (0.5 *μ*g) from REnCs was isolated and purified with a commercial kit (Zymo Research, Orange, CA). The DNA was annealed to primer targets via T4 DNA ligase as previously described (Guo et al. 2004). The ligated DNA template was amplified by PCR for 26 cycles at 94°C (1 min)/72°C (3 min) to better show apoptotic DNA laddering. To standardize the amount of DNA template that was used, standard PCR to the gene *En‐2* was performed with specific primers (data not shown).

### Transendothelial permeability assays

Mouse cells, 3 × 10^5^, were seeded on Transwell filter inserts (Cell Culture Insert 1.0 *μ*m; Falcon, Franklin Lakes, NJ) in 24 multiwell plates and allowed to grow to confluence. The cells were then treated with control media or TNF (20 ng/mL) with or without inhibitors for 24 h. Transendothelial permeability to macromolecules was assessed by measuring passage of FITC‐labeled BSA across the cell monolayer. Briefly, the medium in the insert was replaced with 500 *μ*L of serum‐free medium (SFM) containing 0.125 mg/mL FITC‐labeled BSA. At various time points, 20 *μ*L aliquots were removed from the lower chamber for fluorescence measurements using a Synergy HT Multi‐Mode Microplate Reader (BioTek, Winooski, VT); excitation wavelength 485 nm; detection wavelength 528 nm. The amount of FITC‐BSA passing through the monolayer was calculated by reference to a standard curve. Similarly, transendothelial electrical resistance (TEER) was measured with an EVOM voltmeter (World Precision Instruments, Sarasota, FL), with an apical volume of 500 *μ*L and a basal volume of 700 *μ*L. The background resistance of the filter inserts was subtracted from the cell measurements.

### Immunofluorescence staining and confocal microscopy

Cells grown on glass coverslips were fixed with 3% paraformaldehyde in PBS. Fixed cells were blocked and permeabilized for 15 min with PBS‐containing 1% bovine serum albumin (BSA) and 0.05% saponin. Cells were stained with Alexa fluor 594‐phalloidin or Alexa fluor 488‐phalloidin, or incubated with the primary Ab in 1% BSA overnight at 4°C and with secondary Ab for 2 h at room temperature. For WGA staining in living cells, cells were grown to confluence on glass coverslips and washed with PBS‐containing calcium and magnesium, and thereafter incubated with Alexa Fluor 594‐WGA at 2 *μ*L/mL for 30 min. Cells were mounted in Fluoro‐Gel mounting medium (Electron Microscopy Sciences, Hatfield, PA). Images were collected with a Fluoview 200 laser scanning confocal microscope equipped with a 647‐nm argon laser at ×20 and ×60 magnification. Gap area between cells was measured by a blinded observer on randomly selected 60× fields of each group using ImageJ software (public domain; NIH; imagej.nih.gov), and expressed as percentage of the total area of the field. Intensity of linear ZO‐1 staining was measured in control and TNF‐treated groups with ImageJ in a similar fashion, expressed as a percentage of the control intensity. XZ scan images were captured with a Leica SP5 II AOBS confocal microscope equipped with a 100x/1.46 oil objective (Leica Microsystems, Inc., Buffalo Grove, IL). To quantify cell surface WGA and HSPG expression, densitometric analysis of the intensity of the cell surface fluorescence signals was performed on digitized images of GEnCs using FIJI software (public domain; fiji.sc).

### Protein preparation and immunoblotting

After treatment with or without TNF, EnCs were scraped from plastic six‐well plates in RIPA lysis buffer supplemented with protease inhibitors (Roche Complete Protease Inhibitor Cocktail tablets) at 4°C. Protein concentration for each sample was determined by the BCA method. Forty micrograms of proteins was run in each lane on a 4–12% SDS‐PAGE gel (Invitrogen Nu‐PAGE) and transferred onto Immobilon‐P nitrocellulose membrane. Membranes were then blocked in 5% milk‐TBST and probed with the primary Ab at 4°C. After washing, membranes were incubated for 2 h with secondary Ab (800 nm goat anti‐rabbit IgG; Li‐Cor Biosciences, Lincoln, NE) and the protein bands were detected by an Odyssey infrared imager (Li‐Cor Biosciences, ODY‐1320). Band density was measured with ImageJ and normalized to actin control for each lane.

### RNA isolation and real‐time quantitative polymerase chain reaction (PCR)

Total RNA extraction from cells and reverse transcriptase reactions were performed as described previously (Eadon et al. 2012). Real‐time PCR was performed using the Applied Biosystems 7900 system and the SybrGreen intercalating dye method with HotStar DNA polymerase (Applied Biosystems, Foster City, CA). The ratio of expression of the gene of interest relative to 18S expression was calculated for each sample and normalized to the control group. Synthesis of primers was performed by Invitrogen Custom Primers (Camarillo, CA), with sequences as follows: 18S forward primer 5‐GTT GGT GGA GCG ATT TGT CT‐3, 18S reverse primer 5‐GAA CGC CAC TTG TCC CTC TAT‐3, claudin‐5 forward primer 5‐CCT TCC TGG ACC ACA ACA TC‐3, claudin‐5 reverse primer 5‐GCC GGT CAA GGT AAC AAA GA‐3, claudin‐15 forward primer 5‐GCA GGG ACC CTC CAC ATA C‐3, claudin‐15 reverse primer 5‐GCA CTC CAG CCC AAG TAG AG‐3, claudin‐12 forward primer 5‐ACT GCT CTC CTG CTG TTC GT‐3, claudin‐12 reverse primer 5‐TGT CGA TTT CAA TGG CAG AG‐3, ZO‐1 forward primer 5‐GAC CTT GAG CAG CCG TCA TA‐3, ZO‐1 reverse primer 5‐CCG TAG GCG ATG GTC ATA GTT‐3, Occludin forward primer 5‐TGG CTG CTG CTG ATG AAT A‐3, Occludin reverse primer 5‐CAT CCT CTT GAT GTG CGA TAA T‐3.

### Scanning electron microscopy

Mouse REnCs and human GEnCs were cultured in 24‐multiwell plates on collagen‐coated cover slips, washed with PBS buffer and fixed with 2.5% glutaraldehyde in PBS for 72 h. The samples were then dehydrated in ethanol solutions of 70–100% three times, for 10 min each. The samples were then immersed for 15 min in a 50/50 mixture of ethanol and hexamethyldisilazane (HMDS; Sigma) and then 20 min in 100% HMDS for drying as described (Haseloff et al. 2015). After drying, the samples were mounted on stubs and sputter coated with 80%Pt/20%Pd to 8 nm by Cressington Sputter Coater 208HR. Samples were examined with a Fei NovaNano SEM200 electron microscope (FEI Company, Hillsboro, Oregon) at a distance of 5 mm. The structure of the cells was evaluated and the fenestrae diameters were measured by ImageJ analysis of digitized images.

### Statistical analyses

SigmaStat 3.5 statistical software package (Systat Software, San Jose, CA) was used for all analyses. Data are presented as means ± standard error of the mean, unless otherwise noted. Groups were compared by two‐tailed *t*‐test, or analysis of variance when more than two groups were compared. A value of *P *<* *0.05 was taken to indicate statistical significance.

## Results

### Barrier dysfunction is induced by LPS in mice and by TNF in renal endothelial cells

**Figure 1:**
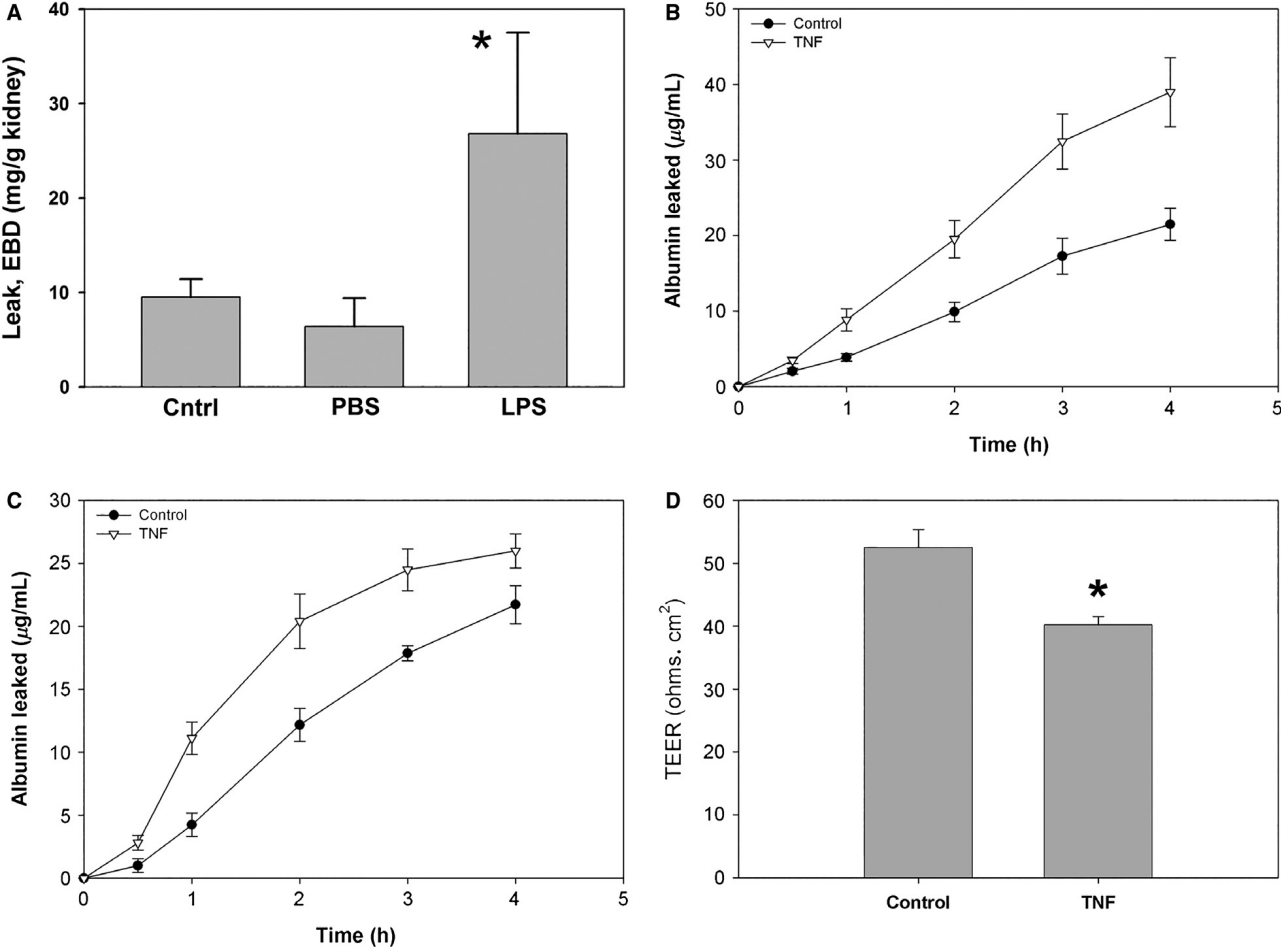
TNF causes barrier dysfunction in human GEnCs and mouse REnCs. (A) Mice injected with Evans blue dye 23 h after LPS showed significant extravascular leakage of dye into the renal parenchyma. (B, C) Cumulative leakage of FITC‐BSA over time in human GEnCs (B) and mouse REnCs (C). Monolayers were cultured under control conditions with or without TNF (20 ng/mL) for 24 h. (D) TNF decreased the transendothelial electrical resistance (TEER) of mouse REnCs. *P <* *0.05 between control and LPS or TNF, *n *=* *3–4 per group for all depicted experiments.

To demonstrate the effect of LPS‐induced AKI on vascular leak inside the kidney, Evans blue dye (EBD) was injected in to mice 23 h after LPS. As seen in Figure 1A, this demonstrates a marked leakage of EBD‐labeled albumin into the renal interstitium compared to PBS. A prime candidate as a mediator for this effect is TNF, which is released in to the circulation soon after LPS injection (Guo et al. 2004). TNF is known to increase permeability of endothelial cells, although this is generally a delayed response (Goldblum et al. 1989; Goldblum and Sun 1990). McKenzie and Ridley (2007) showed that the effect of TNF (10–100 ng/mL) on the permeability of HUVEC monolayers was significant 8 h after its addition, and peaked at 24 h. Here, we tested the effect of TNF on the permeability of human glomerular endothelial cells (GEnCs) and mouse primary culture renal endothelial cells (REnCs), which are mainly a mixture of glomerular and peritubular endothelial cells (Wu et al. 2009). As shown in Figure 1B, exposure of human GEnC monolayers to TNF for 24 h leads to a significant increase in the passage of labeled albumin across monolayers compared with controls (*P* ≤ 0.01 compared to control, *n *=* *5). Exposure of mouse REnC monolayers to TNF for 24 h also lead to a significant increase in the passage of labeled albumin across monolayers compared with controls (*P* ≤ 0.01 compared to control, *n *=* *9, Fig. 1C). To confirm the formation of a tight monolayer, transendothelial electrical resistance (TEER), a measure of ion flux, was obtained by measuring the overall resistance to the current between electrodes across the monolayers using EVOMx voltometer. The mean TEER of mouse REnC monolayers under control condition was 52.5 ± 2.8 Ω•cm^2^ (*n *=* *3) at 37°C, a value compatible to that of published endothelial TEER (Wuest and Lee 2012), whereas TNF treatment significantly decreased TEER to 40.2 ± 1.3 Ω•cm^2^ (*P *<* *0.01; *n *=* *4) (Fig. 1D).

### TNF‐induced barrier dysfunction does not depend on caspase activation

**Figure 2:**
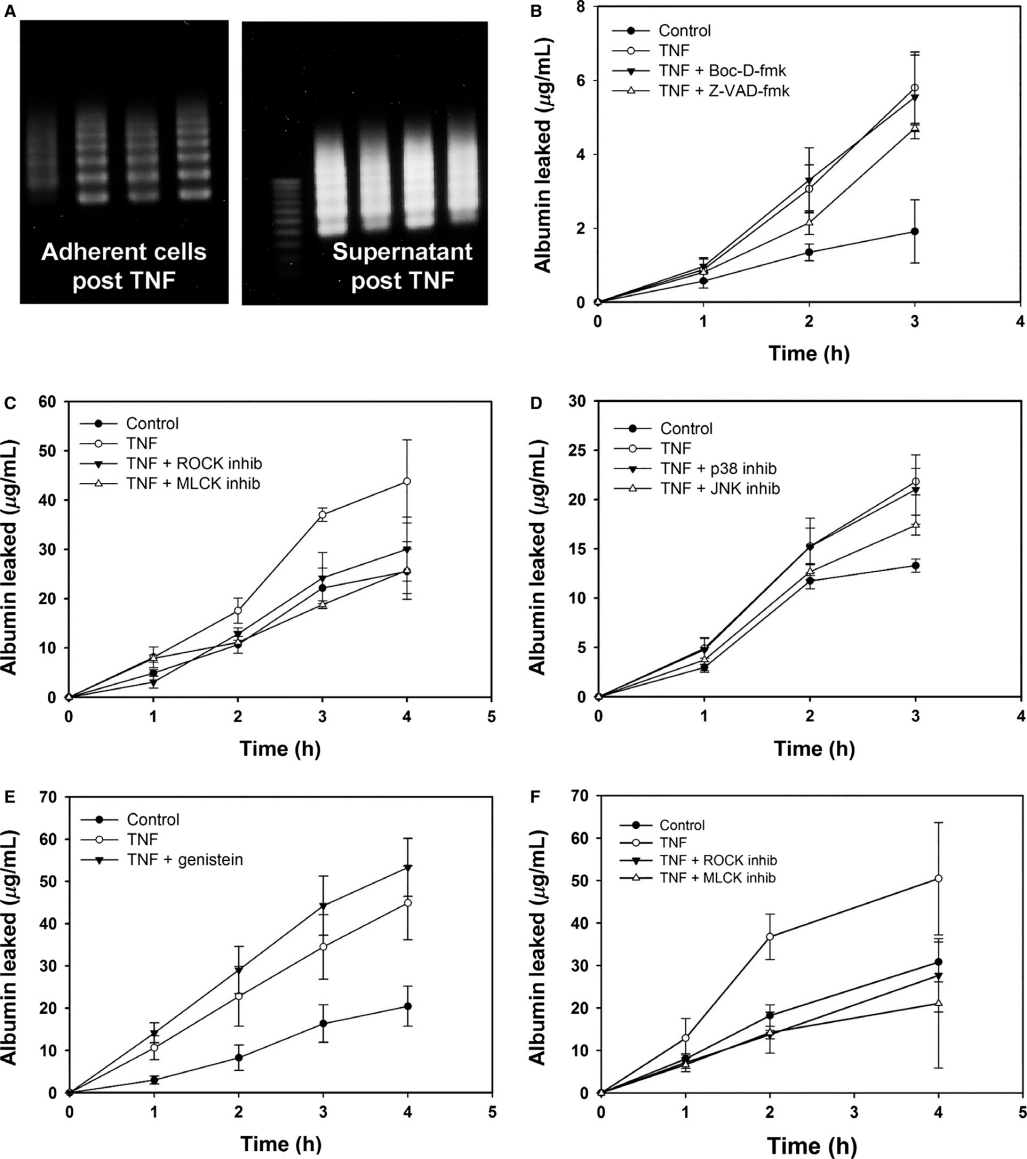
TNF‐induced barrier dysfunction is dependent on ROCK and MLCK. (A) Incubation of mouse REnCs with TNF causes significant apoptosis at 24 h, as shown by the LM‐PCR assay. The extent of apoptosis was significantly greater in the supernatant versus cells that remained attached, showing that apoptotic cells detach. (B) However, neither broad‐spectrum caspase inhibitors Z‐VAD‐fmk nor Boc‐D‐fmk (50 *μ*mol/L each) prevented the TNF‐induced permeability increase. (C) In contrast, preincubation with ROCK inhibitor Y‐27632 (5 *μ*mol/L) or MLCK inhibitor ML‐7 (10 *μ*mol/L) did significantly lessen the TNF‐induced permeability increase. (D, E) JNK inhibitor SP600125 (20 *μ*mol/L) also inhibited the TNF‐induced permeability increase, whereas p38‐MAPK inhibitor SB202190 (10 *μ*mol/L) and tyrosine kinase inhibitor genistein (10 μg/mL) did not. (F) Inhibitors of ROCK and MLCK similarly inhibited TNF‐induced permeability increase in human GEnCs. Inhibitors alone did not affect baseline permeability (data omitted for clarity). Graphs show cumulative leakage of FITC‐BSA over time in REnC monolayers cultured under control or TNF (20 ng/mL) for 24 h before measurement.

Tumor necrosis factor is well known to cause apoptosis through the activation of caspase‐8 (Guicciardi and Gores 2009). We have previously shown apoptosis to occur in renal endothelium in the kidney during endotoxemia, as well as in cultured mouse REnCs (Guo et al. 2004; Wu et al. 2009). Thus, we initially hypothesized that detachment of apoptotic cells from the endothelial monolayer could account for TNF‐induced barrier dysfunction. We did confirm through the ligase‐mediated PCR (LM‐PCR) DNA laddering assay that both human and mouse REnCs undergo apoptosis, with far more apoptosis evident in detached cells collected from the supernatant (Fig. 2A). To test the role of apoptosis in TNF‐induced endothelial barrier dysfunction, we used broad‐spectrum caspase inhibitors, at doses and durations which we have previously shown to block apoptosis in these cell lines (Wu et al. 2009). Surprisingly, however, two different broad‐spectrum caspase inhibitors, z‐VAD‐fmk and Boc‐D‐fmk, both failed to prevent TNF‐induced increase in permeability in mouse REnCs (as shown in Fig. 2B).

### ROCK and MLCK are required for TNF‐induced renal endothelial barrier dysfunction

ROCK and MLCK are the main regulators of actomyosin contractility, and have been shown to be mediators of permeability induced by substances such as thrombin and LPS in other endothelial cell lines (Vandenbroucke et al. 2008). Therefore, we tested the involvement of ROCK and MLCK in TNF‐induced changes to permeability in endothelial cells. Mouse REnC monolayers were incubated with the ROCK inhibitor Y‐27632 together with TNF for 24 h. As shown in Figure 2C, inhibition of ROCK with Y‐27632 abolished the TNF‐induced permeability increase (*P *≤* *0.01 compared to TNF). The MLCK inhibitor, ML‐7, also significantly prevented TNF‐induced permeability increase (*P *≤* *0.01 compared to TNF, Fig. 2C). Neither ROCK inhibitor nor MLCK inhibitor affected the basal permeability of mouse REnCs. The permeability induced by TNF in human GEnCs was similarly blocked by Y‐27632 and ML‐7 (Fig. 2F). These results indicate that ROCK and MLCK play a key role in TNF‐induced increase in permeability in renal endothelial cells.

Mitogen‐activated protein kinase (MAPK) cascades have also been shown to mediate cytoskeletal regulation of endothelial permeability (Yuan 2002; Bogatcheva et al. 2003). MAPKs comprise three major classes: extracellular signal‐regulated kinases (ERK1/2), p38 MAPK, and c‐Jun NH2‐terminal kinase (JNK). These pathways are strongly activated by cytokines such as TNF, both through the TNFR1 signaling complex, and perhaps indirectly downstream of Rho (Mong et al. 2008). To evaluate the involvement of MAP kinases in renal EnC barrier regulation, we employed p38‐MAPK inhibitor SB202190 and JNK inhibitor SP600125, and studied their effect on TNF‐induced albumin leak. As shown in Figure 2D, inhibition of JNK with SB600125 did not abolish, but significantly attenuated, TNF‐induced increase permeability in mouse REnCs (*P *≤* *0.05 compared to TNF), whereas p38 inhibitor SB600125 did not have a significant effect. TNF has also been found to activate tyrosine kinase and subsequently increase albumin leak in human lung endothelial cells (Angelini et al. 2006). However, we found that the tyrosine kinase inhibitor genistein failed to prevent TNF‐induced increase in permeability in mouse REnCs (Fig. 2E). Thus, the TNF‐induced permeability increase in the renal endothelium is dependent on ROCK, MLCK, and to a lesser extent, upon JNK.

### TNF‐induced barrier dysfunction correlates with changes in actin organization and endothelial morphology

**Figure 3:**
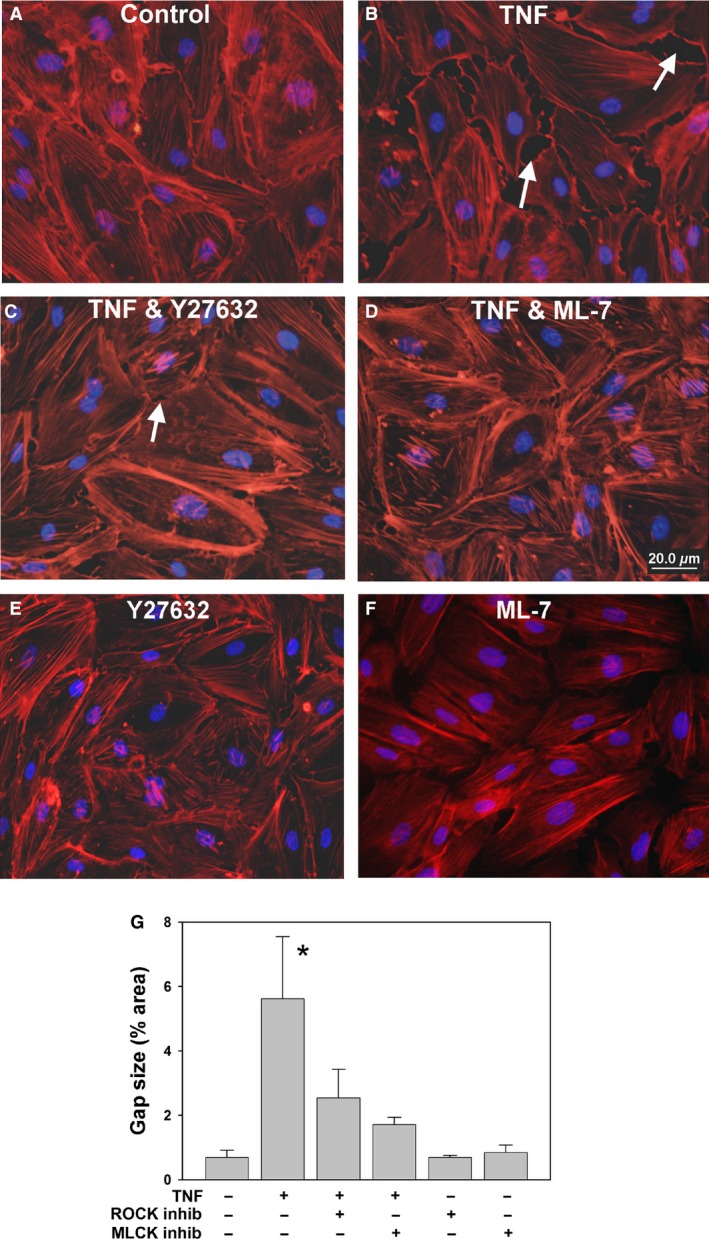
TNF induces stress fibers and intercellular gap formation in a ROCK and MLCK dependent fashion. Human GEnCs were either left unstimulated (A), or were treated with TNF alone (B), TNF + Y27632 (5 *μ*mol/L) for 24 h (C), or TNF + ML‐7 (10 *μ*mol/L) for 24 h (*D*). Single confocal sections of cells stained for F‐actin (red) and DAPI (blue) were imaged, with representative images shown. Intracellular gaps induced by TNF, shown by arrows, were smaller and less frequent in cells pretreated with either Y27632 or ML‐7, as were longitudinal stress fibers. Neither Y27632 nor ML‐7 controls had a notable effect on actin stress fibers (E, F). Scale bar = 20 μm. Measurement of total intercellular gap area in each group is shown in G; *n* = 4 randomly chosen fields per group. *P <* *0.05 versus all other groups.

Given the function of Rho and MLCK in actin‐myosin contractility, confluent human GEnC and mouse REnCs were stimulated with TNF, and fixed and stained for F‐actin. Confocal images were taken to show the F‐actin distribution within cells. As shown in Figure 3, prior to TNF stimulation, the majority of actin filaments in mouse REnCs were localized around the periphery of cells, in parallel with cell–cell junctions. After TNF treatment, most cells were elongated, with thick actin stress fibers that traversed the cells in the direction of cell elongation, with the appearance of gaps at cell–cell junctions (Fig. 3B). As expected, treatment with both ROCK and MLCK inhibitors prevented these changes, restoring a more peripheral actin distribution, decreasing stress fibers, and decreasing intracellular gaps (Fig. 3C, D and G). The TNF induced similar changes in actin organization in human GEnC cells (data not shown). The increase in permeability in renal endothelial cells induced by TNF therefore correlates with the changes in morphology and actin organization, as observed in HUVEC cells (McKenzie and Ridley 2007).

### TNF induces changes to tight junction proteins

**Figure 4:**
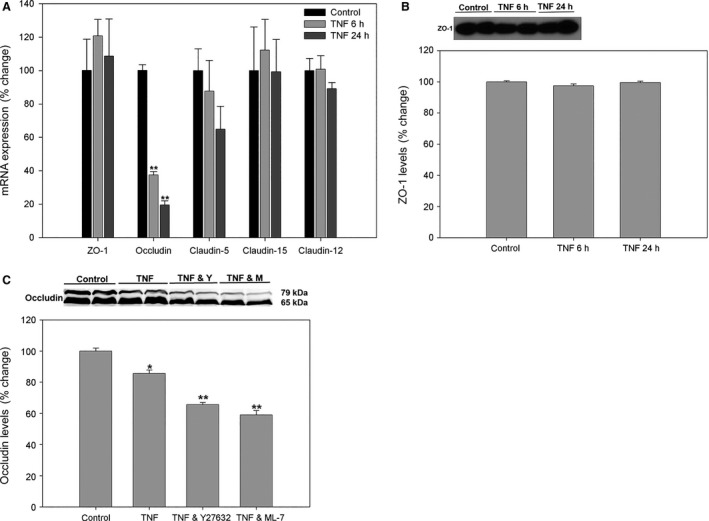
TNF causes changes in tight junction gene and protein expression. (A) Effect of TNF on mouse REnC tight junction mRNA expression, shown as a ratio to 18S. ZO‐1, Claudin‐5, ‐15, and ‐12 mRNA expression did not change significantly after TNF treatment. Occludin mRNA expression was significantly decreased at 6 and 24 h. (B) Western blots show the total amount of ZO‐1 in cell lysate was not changed by TNF. Band densities are expressed as a ratio to actin. (C) Western blots showing the amount of occludin in cell lysate significantly decreased at 6 and 24 h after TNF. Both the known heavier occludin band (>70 kDa) and the lower molecular weight band (approximately 65 kDa) decreased. Protein or gene expression in control cells was defined as 100%. *P <* *0.05 versus control. **P <* *0.01 versus control, <0.05 versus TNF alone group.

To identify changes to tight junctions that might contribute to the increase in permeability, the tight junctional protein ZO‐1 was examined after TNF exposure. The TNF induced no significant changes in global ZO‐1 protein level measured by RNA expression (Fig. 4A) or Western blotting (Fig. 4B) in mouse REnCs. We also examined the protein and RNA expression of other endothelial tight junctional proteins occludin, claudin‐5, claudin‐12, and claudin‐15 in these mouse REnCs 6 and 24 h after TNF exposure. These claudins have variably been found to be expressed in renal endothelium, including the kidney glomerulus (Morita et al. 1999; Inai et al. 2005; Koda et al. 2011). These tight junctional proteins failed to consistently stain cell–cell junctions by immunofluorescent microscopy in mouse REnCs and human GEnCs (data not shown). The mRNA expression of claudin‐5, ‐12, and ‐15 did not change significantly after TNF exposure. Occludin mRNA expression decreased at 6 and 24 h after TNF exposure (37.5 ± 1.9% at 6 h, *P *<* *0.01: 19.5 ± 2.5% at 24 h, *P *<* *0.01, Fig. 4A). Similarly, 24 h after TNF treatment, occludin protein levels were significantly decreased on Western blot (Fig. 4C). However, this TNF‐induced decrease in overall levels of occludin protein was actually worsened by ROCK inhibitor Y‐27632 and the MLCK inhibitor ML‐7 (Fig. 4C), opposite of the effect of these inhibitors on permeability.

**Figure 5:**
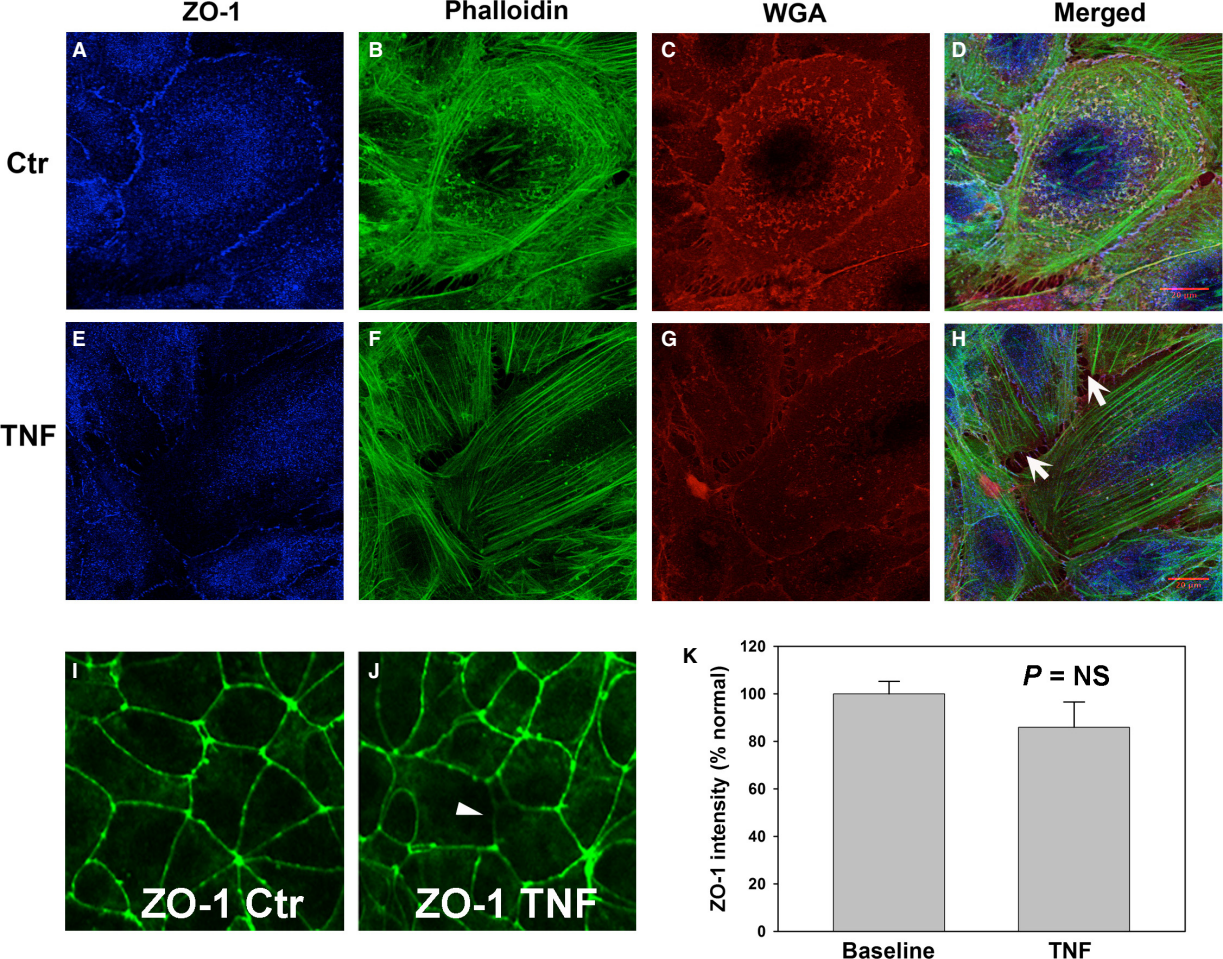
TNF disrupts ZO‐1 expression in the tight junctions of human GEnCs. Confocal images of endothelial monolayers show occasional moderate disruption of submembrane ZO‐1 in TNF‐treated cells (A, E), occurring simultaneously with actin redistribution from the cell periphery to longitudinal stress fibers (B, F). Likewise, WGA staining was significantly decreased by TNF treatment (C, G). Merge of these images is shown (D, H); intracellular gaps are indicated by arrows. Scale bar = 20 *μ*m. (I–K) However, ZO‐1 distribution was mostly unaffected by TNF, with only focal disruption (arrowhead). (K) Quantitation of submembrane ZO‐1 staining was not significantly affected by TNF.

Confocal images of cells costained for F‐actin, WGA and ZO‐1 were taken at the level of the tight junctions (Fig. 5), to demonstrate the spatial relationship of these changes. At baseline, ZO‐1 formed a continuous line at cell–cell borders. The majority of F‐actin was localized in parallel with the cell periphery and some thin lines of F‐actin colocalized with ZO‐1 (Fig. 5A and B). After TNF treatment, ZO‐1 presence at cell–cell junctions was focally decreased, and its distribution was somewhat fragmentary (Fig. 5E, I and J), indicating that tight junction integrity was partially disrupted, with more gaps formed at cell–cell junction (arrows in Fig. 5H). However, quantitation of linear submembrane ZO‐1 density from randomly selected fields showed that this difference did not reach statistical significance (Fig. 5K).

### TNF disrupts the glycocalyx in renal endothelial cells

**Figure 6:**
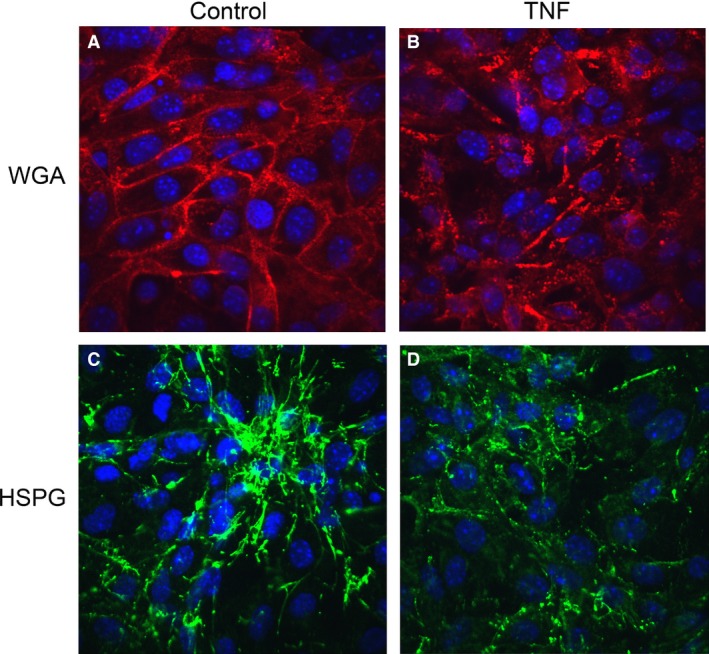
TNF disrupts the renal endothelial glycocalyx. (A, B) Confocal laser scanning microscopy images of living mouse REnCs labeled with WGA‐Alexa594 lectin (red) and counterstained with DAPI (blue) show disruption and reduction in the glycocalyx after exposure to TNF for 24 h. (C, D) Confocal laser scanning microscopy images of mouse REnCs labeled with anti‐HSPG (green) and DAPI show a similar difference.

**Figure 7:**
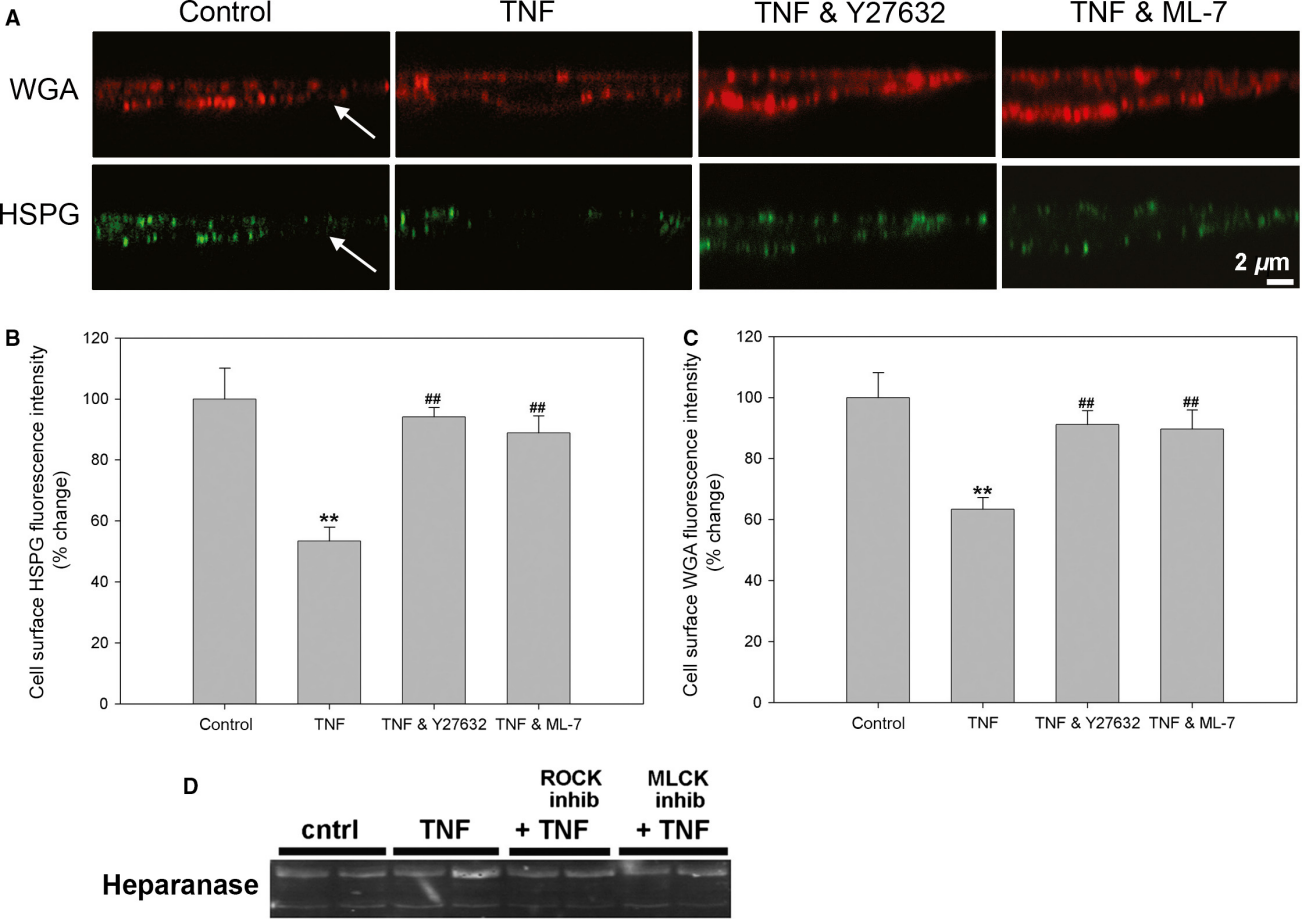
TNF causes degradation of the human GEnC glycocalyx in a ROCK and MLCK dependent fashion. (A) Confocal x‐z plane reconstructions showing cell surface immunofluorescence of WGA (red) and HSPG (green) in human GEnCs are shown. Cells were either left untreated (control), or treated with TNF for 24 h, with or without inhibitors of ROCK (Y27632, 5 *μ*mol/L), or MLCK (ML‐7, 10 *μ*mol/L). Arrows indicate cell apical surface. Scale bar = 2 *μ*m. (B, C) Cell surface HSPG and WGA fluorescence intensity was measured for each cell by measuring densitometry of a line drawn along the cell edge. TNF caused a decrease in both cell surface HSPG and WGA staining, which was significantly prevented by inhibition of either ROCK or MLCK. (D) Western blot for heparanase showed no significant changes after TNF, with or without inhibitors. Intensity normalized to control are expressed (*n *=* *26–36 sections for each group). **P <* *0.01 versus control; ##*P *<* *0.01 versus TNF alone group.

The luminal aspect of all endothelia is covered by a mesh‐like hydrated structure known as glycocalyx. Singh et al. (2007) showed that the apical glycocalyx constitutes a barrier to protein in cultured human glomerular cells. It has been suggested that TNF disrupts the glycocalyx covering the intact hamster cremaster microcirculation (Henry and Duling 2000). Here we tested whether TNF disrupts the glycocalyx layer in GEnCs, as determined by binding of wheat germ agglutinin (WGA)‐Alexa594, which binds to sugar residues in glycoproteins present in glycocalyx in unfixed cells, as well as by immunostaining for heparan sulfate proteoglycan (HSPG), a major component of the glycocalyx (Singh et al. 2007). Confocal images of renal EnCs costained for WGA or HSPG taken at the level of the cell nucleus showed that the intact cell surface distribution of glycocalyx (Fig. 6A) was disrupted by TNF treatment (Fig. 6B). Immunoconfocal studies also uncovered abundant HSPG coating mouse REnCs, which was greatly decreased after TNF treatment (Fig. 6C and D). To supplement these horizontal plane images, we studied the glycocalyx height by vertical (XZ) laser scanning confocal microscopy techniques, which gave an index of glycocalyx volume on the endothelial apical surface (Fig. 7). The XZ scanning showed that TNF treatment significantly decreased the cell surface WGA and HSPG abundance in human GEnCs (Fig. 7A), from 100.0 ± 10.2% to 53.4 ± 4.6% (*P *<* *0.01). Treatment with ROCK inhibitor Y27632, as well as MLCK inhibitor ML‐7, significantly prevented HSPG degradation (*P *< 0.01, Fig. 7B). Similarly, TNF treatment decreased surface WGA staining from 100.0 ± 8.2% to 63.3 ± 3.9% (*P *<* *0.01), which was prevented by either ROCK or MLCK inhibitors (Fig. 7C).

One possible mechanism by which inflammation, such as TNF, could degrade the glycocalyx is by increased expression or activation of heparanase, as has been seen in the mouse glomerulus in models of sepsis (Lygizos et al. 2013; Xu et al. 2014). Although, we found heparanase was expressed in these cells, we did not see upregulation after TNF or any change with ROCK or MLCK inhibitors, either by Western (Fig. 7D), or by RT‐PCR. Another possibility is that TNF causes increased expression of matrix metaloproteinase‐9 (MMP‐9), leading to glycocalyx degradation (Ramnath et al. 2014). However, in our mouse and human renal EnCs, we found minimal expression of MMP‐9 at the protein level (data not shown).

### TNF alters the appearance of fenestrae in glomerular endothelial cells

**Figure 8:**
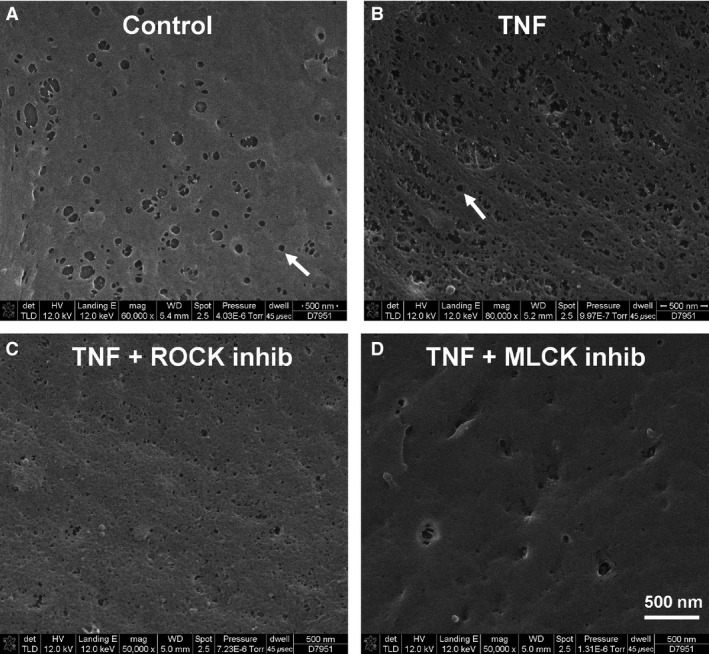
TNF causes smaller fenestrae size in human GEnCs. Demonstration of fenestrae in human GEnCs by SEM; representative images of four separate experiments are shown. Arrows indicate two fenestrae with a diameter of about 80 nm. Representative images show TNF‐treated cells (B) have smaller but more numerous fenestrae compared to untreated control cells (A). Preincubation with ROCK inhibitor Y27632 with TNF caused a reduction in the number of fenestrae, but their size remained small (C). Preincubation with MLCK inhibitor ML‐7 restored fenestrae size and number close to that of baseline controls (D). Alternatively, smaller holes (10–50 nm) may represent pinocytotic vesicles. Scale bar = 500 nm.

A key pathway through which small solutes pass through the glomerular endothelium in vivo is fenestrae, transcellular channels of approximately 60–100 nm size. Although this is significantly wider than an albumin molecule, the fuzzy glycocalyx layer significantly decreases the effective diameter of the fenestrae (Deen et al. 2001; Haraldsson et al. 2008). In a previous publication, we have shown that glomerular fenestrae are dramatically enlarged, although less numerous, after injection of either TNF or LPS into mice (Xu et al. 2014). To determine a possible role for fenestrae in accounting for the permeability changes we described, we performed scanning electron microscopy to visualize fenestrae in the human GEnCs. As seen in Figure 8, the cultured human GEnCs did indeed contain cytoplasmic pores suggestive of fenestrae, with a diameter roughly between 60 and 200 nm. Several other types of endothelial cells in the kidney, including those of the peritubular capillaries and ascending vasa recta also contain fenestrae, although the descending vasa recta do not (Pallone et al. 1994). Scanning electron microscopy (SEM) of the mouse REnCs also demonstrated the presence of fenestrae; while the origin of these primary culture mouse REnCs is likely heterogenous, their strong TNFR1 staining (data not shown) suggests they are largely glomerular or peritubular in origin. After TNF treatment for 24 h, human GEnCs became less flat and more contracted, with an apparent increase in number of fenestrae. Simultaneously, the fenestrae diameter decreased (Fig. 8B). The average diameter of the endothelial fenestrae in control human GEnCs was significantly larger than that in TNF‐treated cells (99.4 ± 8.7 vs. 48.33.4 nm, *P *<* *0.01). Coincubation with MLCK inhibitor ML‐7 and TNF restored fenestrae size and number close to that of baseline controls (Fig. 8D). Use of ROCK inhibitor Y27632 with TNF also caused a reduction in the number of fenestrae, but interestingly, their size remained small (Fig. 8C). An alternative or overlapping possibility is that the changes seen on SEM after TNF indicate an increase in pinocytosis, which typically has smaller vesicles of approximately 10–50 nm in size (Fig. 8B and C).

## Discussion

Prior work in animal models has shown that the cytokine TNF plays a key role in LPS‐induced AKI (Knotek et al. 2001; Cunningham et al. 2002). The predominant receptor for TNF, TNFR1, is expressed mainly on leukocytes, but within the kidney has strongest expression in the peritubular and especially the glomerular endothelium (Al‐Lamki et al. 2001). TNF induces inflammatory signaling in the endothelium, causing expression of chemokines, and adhesion molecules such as ICAM‐1 (Wu et al. 2007). Another manifestation of inflammation, vascular leak, is prominent in sepsis, leading to events such as pulmonary edema or septic encephalopathy (Alexander et al. 2008). Here, we examine possible mechanisms for the increase in permeability induced in the renal endothelium caused seen in sepsis.

In this report, we confirm that TNF induces an increase in permeability in both mouse and human renal endothelium. This increase in permeability in mouse REnCs and human GEnCs was strongly dependent on ROCK and MLCK. Rho is an important pathway through which diverse stimuli cause an increase in permeability in epithelial and endothelial monolayers. Activation of Rho leads to activation of ROCK, which inhibits dephosphorylation of myosin light chain, in turn leading to rearrangement of the actin cytoskeleton (Petrache et al. 2003). Blocking these pathways minimized gaps between EnCs in parallel with preservation of barrier function. It must be noted that inhibition of the Rho pathway is quite potent in preventing actin stress fiber formation in favor of cortical actin distribution, which could possibly obscure other more subtle mechanisms through which TNF influences permeability. Increased permeability is often associated with loss or redistribution of tight junction proteins, for example a loss of occludin from tight junctions after TNF stimulation in HUVEC cells (McKenzie and Ridley 2007). We did observe subtle changes to expression of tight junction proteins, such as occludin and ZO‐1. Interestingly, the shift toward lower molecular weight occludin isoforms that we saw has been noted before in intact kidney after LPS injection (Eadon et al. 2012). However, these changes in tight junction proteins are unlikely to be the primary or sole explanation for TNF‐induced renal EnC barrier dysfunction, since they were relatively modest, weaker than in other endothelial cell lines, such as HUVECs (McKenzie and Ridley 2007). Also, while inhibitors of ROCK and MLCK preserved barrier function, they actually worsened total occludin expression. It may simply be that actin‐driven changes in the cytoskeleton causing the simple physical separation and rounding up of cells, as suggested on electron microscopy, is responsible for much of the changes in permeability induced by TNF.

It is well established that renal endothelial cells undergo apoptosis in response to high concentrations of TNF, mediated through caspase‐8, via physical interaction with the large and complex TNFR1 signaling complex (Guicciardi and Gores 2009). While one may imagine that apoptotic endothelial cells leave an opening in the endothelial barrier layer, we surprisingly found that caspase inhibition did not preserve barrier function downstream of TNF. It may be possible that neighboring endothelial cells quickly clear apoptotic bodies and/or fill such gaps (Petrache et al. 2001). In a previous report, we suggested that Rho activation is dependent in part upon caspase activation (Wu et al. 2009); however the fact that Rho‐dependent permeability increases were not blocked with two different caspase inhibition calls these previous findings into question. Our results suggest that JNK activation downstream of TNFR1 may play a partial role in permeability changes, as has been reported in other endothelial cells (Fahmy et al. 2006), while activation of p38 MAPK or tyrosine kinases showed no significant effect.

Recently, it was reported that TNF caused degradation of the glycocalyx component HSPG in immortalized human glomerular endothelial cells, accompanied by an increase in the permeability to albumin (Ramnath et al. 2014). HS core protein expression was detected in the supernatant, suggesting shedding of HS proteoglycan in response to TNF, although decreased synthesis of endothelial proteoglycans could also play a role. We found similar results in mouse REnCs and human GEnCs, and are the first to report that glycocalyx degradation is dependent on ROCK and MLCK. Ramnath et al. found evidence that matrix MMP‐9 was responsible for TNF‐induced endothelial shedding of HSPG, which is intriguing given that MMP‐9 expression has been previously found to be ROCK dependent (Jeong et al. 2012). However, we were unable to confirm significant expression of MMP‐9 in our cells. As an alternative mechanism, microvascular endothelial cells secrete heparanase under inflammatory conditions, an endo‐beta‐d‐glucuronidase that specifically cleaves the heparan sulfate chain of HSPGs. Although we and others have detected an increase in glomerular heparanase expression in animal models of septic AKI (Lygizos et al. 2013; Xu et al. 2014), we failed to detect any significant changes in heparanase levels in our cell model. Other enzymes such as hyaluronidase may potentially play a role in glycocalyx degradation as well. Thus, the mechanism of glycocalyx degradation downstream of TNF and Rho may differ in separate model systems, and will require further work to fully explain.

Renal glomerular endothelial cells have nondiaphragmed fenestrae, which are small transcellular pores with diameters of 60 to 80 nm in vivo, involved in the exchange of molecules across the GFB (Haraldsson et al. 2008; Satchell and Braet 2009). These fenestrations occupy 20–50% of the endothelial surface in the glomerulus. Whether TNF affects glomerular endothelial cell fenestration in vitro has not been previously studied. We found that following treatment with TNF, GEnC fenestrae diameter significantly decreased. How TNF changes glomerular endothelial fenestrae organization is not known. These findings are consistent with those of Yokomori et al. (2004), who found that the fenestrae in liver sinusoidal endothelium became smaller under conditions of pharmacologic Rho activation, which implies the actin cytoskeleton is involved in fenestral morphology. Given that Rho is activated by TNF in REnCs (Wu et al. 2009), it is possible that the effect of TNF on human GEnC fenestrae organization is exerted through Rho activation. Consistent with this, we found that inhibition of MLCK prevented these TNF‐induced fenestrae changes; inhibition of Rho kinase also restored the number of fenestrae toward baseline, however their individual size remained small. Fenestrae are very challenging to study in cell culture models, in part because, unlike podocyte slit diaphragms, there are no known proteins specifically associated with fenestrae (Satchell and Braet 2009). Additionally, all cell culture experiments are by definition artificial; our findings in these cultured glomerular EnCs are actually opposite to what we found by transmission EM in mouse kidney, where TNF or LPS injection each led to fewer, but larger fenestrae (Xu et al. 2014). The net effect of these changes in fenestrae upon permeability to large (or small) molecules is difficult to determine at this point, and awaits further work. Given our other finding that TNF causes degradation of the glycocalyx and intracellular gap formation, it may be premature to conclude that these smaller, more numerous fenestrae also explain TNF‐induced permeability increase.

The role of endothelial barrier dysfunction within the kidney is likely complex. AKI is not a proteinuric disease, but given the reduced GFR in this setting, the fractional filtration of albumin that has been described is not trivial (Xu et al. 2014). The effects we have described in vivo and now in vitro may have greater relevance to diseases such as glomerulonephritis, where localized cytokine expression may accelerate proteinuria through endothelial injury. Changes in fenestrae morphology may conceivably contribute to the decline of GFR in AKI, but confirmation of this requires further work. Perhaps more importantly, excessive vascular leak in the peritubular capillaries in the setting of sepsis could prove injurious via RBC sludging and reduction in perfusion to the renal tubules (Molitoris 2014), and/or by leading to edema and increased interstitial pressure within the renal interstitium.

In summary, TNF increases renal microvascular permeability in a Rho and MLCK dependent fashion, which illustrates the key importance of the cytoskeleton in endothelial injury. These pathways may increase renal endothelial permeability by causing separation between cells and by a loss of the cell surface glycocalyx. The relative role of the different events which we have described remains to be determined. While the activation of Rho and MLCK in diverse endothelial cell subtypes has been described by many investigators, the exact manner in which these pathways are coupled to TNF signaling remains obscure. It is hoped that better insight in to these processes may lead to insights as to the pathogenesis of AKI, sepsis, and other renal diseases.

## Conflicts of Interest

None declared.
